# The start of lightning: Evidence of bidirectional lightning initiation

**DOI:** 10.1038/srep15180

**Published:** 2015-10-16

**Authors:** Joan Montanyà, Oscar van der Velde, Earle R. Williams

**Affiliations:** 1 Universitat Politècnica de Catalunya, Electrical Engineering Department, Barcelona, 08034, Spain; 2 Massachusetts Institute of Technology, Parsons Laboratory, Cambridge, 02139, USA

## Abstract

Lightning flashes are known to initiate in regions of strong electric fields inside thunderstorms, between layers of positively and negatively charged precipitation particles. For that reason, lightning inception is typically hidden from sight of camera systems used in research. Other technology such as lightning mapping systems based on radio waves can typically detect only some aspects of the lightning initiation process and subsequent development of positive and negative leaders. We report here a serendipitous recording of bidirectional lightning initiation in virgin air under the cloud base at ~11,000 images per second, and the differences in characteristics of opposite polarity leader sections during the earliest stages of the discharge. This case reveals natural lightning initiation, propagation and a return stroke as in negative cloud-to-ground flashes, upon connection to another lightning channel – without any masking by cloud.

A key model of a bidirectional lightning leader was introduced by *Kasemir*^1^ in 1960. In his model, leaders grow simultaneously in opposite direction from two sides of a ‘nucleus’ immersed in an electric field. The two leader ends carry charge of opposite polarity by virtue of electrostatic induction in the ambient electrostatic field. The leader is simulated as a prolate spheroid with constant potential and zero net charge. This model has been applied to explain physical development of lightning flashes initiated artificially and naturally^2^,^3^,^4^,^5^,^6^,^7^,^8^, as a basis for 3D lightning models^9^,^10^,^11^ and to address some aspects of the polarity asymmetry of lightning^12^,^13^. Since intra-cloud flashes are nearly always visually masked by clouds, the remote sensing of electromagnetic fields has been typically used^2^,^3^,^14^,^15^,^16^,^17^,^18^. But the weak RF radiation in the propagation of positive leaders complicates the precise mapping of the positive leader development^2^. Only recently, positive leader development has been precisely mapped by Lightning Mapping Array (LMA) networks^19^,^20^,^21^. Direct measurements of current in natural intra-cloud lightning discharges are almost impossible and, up to now, the best opportunity is to measure it by means of instrumented aircraft^22^,^23^ or by triggering by rockets. Reports of artificially triggered lightning by rockets and aircraft indicated that the positive leader started 3–6 ms before the negative leader^2^,^24^,^25^,^26^ presumably because threshold electric fields for positive streamers are less than for the negative counterpart^12^ and the object emitting the positive leader can store negative charge.

## Results

**Figure 1:**
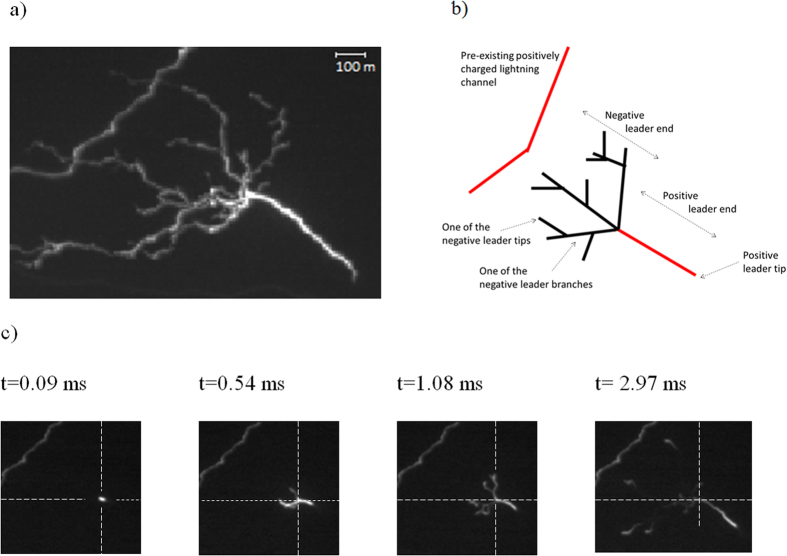
(**a**) Successive video frames of the bidirectional leader before the attachment. The scale is estimated by means of the distance from the observation to the IC detections by the operating VHF interferometer network and the field of view of the camera. (**b**) Sketch of the bidirectional leader and the pre-existing lightning channel. (**c**) Four selected frames at the indicated times. Dashed lines intersect at the origin of the bidirectional leader. For the original video refer to the supplementary information.

On the 9^th^ of September 2012 an electrically unusual storm for this geographic region occurred in the Ebro Valley in northeastern Spain. The storm was prolific in generating positive cloud-to-ground (+CG) lightning flashes (which transfer net positive charge to ground, in contrast to the common –CG flashes). The CG events were recorded with a high speed video camera and confirmed by two lightning detection networks. The frame rate of the camera was adjusted to produce one frame every 90 μs (11,019 images per second). During one recording of an intracloud (IC) flash, a separate bidirectional leader tree is observed to form beneath the cloud base as an independent part of an already ongoing lightning discharge (Fig. 1a). Figure 1b sketches the event representing the bidirectional leader occurring close to a pre-existing lightning channel which had grown ~100 ms before. Its continuous luminosity is suggestive of a long-lasting current in the channel.

The tree form of the bidirectional leader (branches and trunk) shows strong asymmetry in the morphology between its two ends. The bidirectional leader took the form of a tree and not as a double-ended tree as is commonly observed with VHF mapping systems^5^,^10^ for flashes within the cloud. Here it must be noted that only the first 6 ms are discussed, while branching of the positive leader did occur in later stages. Polarities of the bidirectional leader ends are assumed from the characteristics of positive and negative leaders reported by means of high speed video and VHF mapping^27^,^28^,^29^. The left-hand end of the bidirectional leader tree (Fig. 1) exhibits the typical properties of negative leaders observed in high-speed imaging: a high degree of branching, visibility of branches most of the time, and bright leader tips compared to the leader channel left behind. On this basis, the right-hand side of the tree is assumed to be positive. The inferred positive polarity of the right-hand side of the bidirectional leader is also supported by its smaller leader speed, the lack of branches (for the first ~5.3 milliseconds) and the occurrence of recoil leader processes in its later stage. It also has a brighter tip with occasional pulsing, and a brighter leader channel. The polarity of the pre-existing lightning channel was determined to be positive because the negative polarity branch of the bidirectional leader attached to it and by virtue of its lack of branching during its growth. The negative leader branches of the left side of the bidirectional leader all bear the same polarity and do not interact. The same consistent repulsive behavior is observed in discharge ‘trees’ within solid dielectric materials^30^.

**Figure 2:**
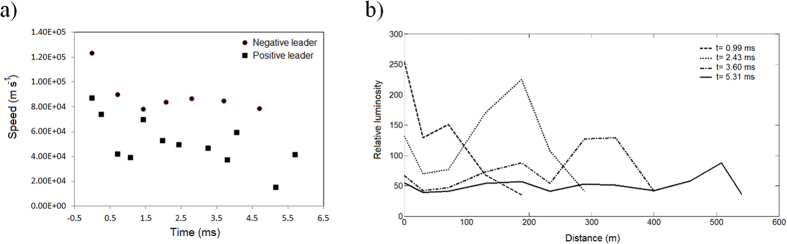
(**a**) Estimated speed of the tips of the positive and negative leader ends; (**b**) Luminosity along the positive leader end at four different times.

Figure 1c displays four frames selected from the 6.08 ms sequence before the negative leader end attached to a pre-existing IC channel charged with opposite polarity. The bidirectional leader started from a bright spot with no appreciable delay in the onset of the positive and negative ends. If some delay took place between the positive and the negative ends it must have been within the time interval between consecutive frames (90 μs to 180 μs). The maximum luminosity was produced during the initiation part and progressively decreased as the bidirectional leader increased in size. On average, the positive leader end was brighter than the negative. Both leader ends presented their maximum speeds during the first millisecond. The positive leader tip speed fluctuated and after 6 ms it decreased to one quarter of the initial speed. On the negative leader side, several tips from branches advanced at the same time. But, since not all the negative branches were visible all the time, their average speed is calculated and represented in Fig. 2a. The speed of the negative leader end remained more steady after the first millisecond and remained larger than the positive leader end by a factor greater than 2.

During the extension of the bidirectional leader, the positive end exhibited luminosity along its channel during its lifetime. The luminosity of the positive leader channel decreased to about one half its initial value just beyond the initiation point. On the other end, for most of the time, only the tips of the negative leader end were visible. In both leader ends, the tips were brighter than the channels behind, similar to previous observations of positive and negative leaders^28^,^31^,^32^. Figure 2b displays the luminosity along the positive leader channel at four different times. It can be noted that the positive leader tip was always brighter than the leader channel behind but the tip brightness decayed as the leader elongated.

**Figure 3:**
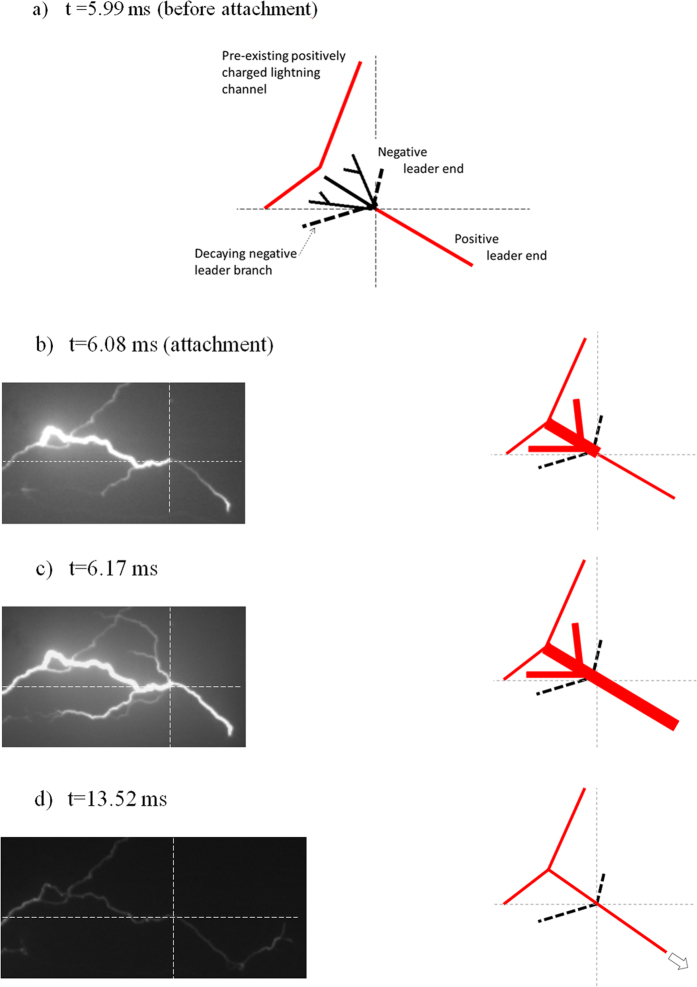
Attachment of the bidirectional leader to the pre-existing lightning channel. Each sketch on the right is a schematic representation of the real camera images on the left. (**a**) t = 5.99 ms: sketch of the bidirectional leader and the pre-existing leader channel before the attachment. Black dashed lines represent decaying branches of the negative leader end that did not appear during the attachment. (**b**) t = 6.08 ms: Attachment between the pre-existing lightning channel and one of the negative leader branches. The attached branch is illuminated up to the initiation point of the bidirectional leader. (**c**) t = 6.17 ms: The illumination is extended to the positive leader end and some of the negative leader end branches. (**d**) t = 13.52 ms: the bidirectional leader acquired positive polarity and advanced as one branch of the pre-existing lightning channel. Thin dashed lines are centered at origin of the bidirectional leader.

For the first 2 ms the negative leader end of the bidirectional leader produced a new branch every ~300 μs. When one of the negative leader branches attached to the pre-existing lightning channel, at t = 6.08 ms (Fig. 3b), one of the branches of the negative leader end was instantaneously illuminated up to the original bidirectional leader initiation point. The next frame (Fig. 3c, t = 6.17 ms) showed re-illumination of some of the other negative leader branches extending up to and then beyond the positive leader end. After that moment, the positive leader end continued advancing as part of a branch attached to the pre-existing lightning channel (e.g. Figure 3d at t = 13.52 ms).

## Discussion

This bidirectional initiation occurred below the cloud as part of a longer lasting flash. Unlike secondary bidirectional leaders known as recoil processes which form commonly within decaying positive leader channels, this case involves new electric breakdown in virgin air, much like the very start of most lightning flashes. In this case the necessary electric field reached the breakdown threshold in the presence of a pre-existing, continuously luminous positive lightning channel. Note that a similar event can be found in images presented by Warner *et al.*^33^, which however was not discussed.

In contrast to studies of rocket-triggered lightning and lightning initiated by aircraft^2^,^24^,^25^,^26^, which found delays of 3–6 ms between the onset of negative and positive leader branches, this natural event exhibited a delay of less than 90 μs (i.e. one video image). Qualitatively, several factors could influence the simultaneous onset of the leaders in a bidirectional leader event. In the case presented, there is no metallic electrode from which the discharge starts (e.g. the wire in the case of rockets, or the aircraft itself). Such an electrode can be polarized and maintain zero net charge when only one leader is progressing in initial stages.

Prominent differences in luminosity between the positive and the negative leader channels are found in the initial bidirectional leader development. The positive leader end was brighter and remained visible during the event whereas the negative leader end was weaker along the channels and their tips were fluctuating in brightness. The tips of the negative leader end were visible almost all the time but after some extension, the negative leader channels became dim and often invisible. That difference is attributable to the ionization processes responsible for the development of the leaders occurring at the tip of the leader. In negative leaders the step propagation implies abrupt current pulses at the tip and intense energy release^8^. The brightness of the leader tips decayed in time as the leader progressed. We assume that the luminosity is related to the current^34^ and references therein. The irregular distribution of the luminosity suggests strong differences in the channel currents. High-time-resolution optical spectra could provide information to estimate leader temperature and thus currents. In the basic bidirectional leader model the charge distribution along the leader channels is assumed to be caused by induction in the ambient electric field and the condition of zero net total charge^1^. *Kasemir*^1^ treated a symmetrical extension, but the observed luminosity and speed differences in our event motivated an asymmetrical treatment^13^.

The attachment of the bidirectional leader to a pre-existing lightning channel led to a brightening of the connecting negative leader branch, similar to the return stroke in a –CG flash upon the negative leader reaching ground. It revealed the uniformity of the polarity of the negative leader end up to the initiation point (Fig. 3a t = 6.08 ms). Indeed, half of the bidirectional leader had different polarity and the neutral point retained its identity.

In summary, this unique observation of “virgin” bidirectional leader development exhibited the following characteristics: i) The positive and negative leader ends of the bidirectional leader began simultaneously within the 90–180 μs time resolution available; ii) the positive leader end was formed by a single channel while the negative leader end was highly branched; iii) the single positive leader channel was visible all the time and was brighter than the multiple negative leader branches; iv) at both leader ends the leader tips were brighter but their luminosity decreased as the bidirectional leader expanded; v) the speed of the positive leader end was a factor of two lower than the average speed of the negative leader end; vi) both ends of the bidirectional leader exhibited their highest speeds during the first millisecond; vii) after the first millisecond, the speed of the positive leader end decreased faster than the negative leader end; viii) at the described attachment, the re-illumination of a negative leader branch up to the origin, allows one to assume that the neutral point of the bidirectional leader remained fixed; ix) the immobility of the neutral point implies that the charge density deposited at the positive leader tip be higher than the charge at the negative leader tips in order to compensate the speeds of the leader ends.

## Methods

Video images were obtained with a Vision Research Phantom v7.3 high speed video monochrome camera with a 28 mm/2.8 lens. The spectral sensitivity of the camera is ~30% QE for the range between 450 nm to 650 nm and between 30% to 20% QE for the range from 650 nm to 750 nm. The frame rate was 11,019 frames per second with an exposure of 88 μs and a resolution of 576 × 480 pixels with 14 bits of information per pixel. Each frame is time stamped by means of a GPS with IRIG-B generator.

## Additional Information

**How to cite this article**: Montanyà, J. *et al.* The start of lightning: Evidence of bidirectional lightning initiation. *Sci. Rep.* **5**, 15180; doi: 10.1038/srep15180 (2015).

## Supplementary Material

Supplementary Information

Supplementary Video
