# Supplementary material for: The start of lightning: Evidence of bidirectional lightning initiation
Source: Sci Rep. 2015 Oct 16;5:15180. doi: 10.1038/srep15180 (PMC4607992; doi:10.1038/srep15180)
Supplement: Supplementary Information [file srep15180-s1.pdf]

Supplementary Material for:

The start of lightning: Evidence of bidirectional lightning initiation

Joan Montanyà[1,*], Oscar van der Velde[1], Earle R. Williams[2]

[1]*Universitat Politècnica de Catalunya, Electrical Engineering Department, Barcelona, 08034, Spain.*

[2]*Massachusetts Institute of Technology, Parsons Laboratory, Cambridge, 02139 ,USA.*

**Correspondence to*: montanya@ee.upc.edu

1. High speed video observation of a bidirectional lightning leader

High speed video at 11,019 images per second of the observed bidirectional lightning leader event. The video shows the initiation and development of the bidirectional leader occurring at the proximity of a pre-existing lightning channel.
